# Geometry‐Regulated Electrode‐Wide Nucleation Patterns in Aqueous Zinc Anodes

**DOI:** 10.1002/advs.77286

**Published:** 2026-09-06

**Authors:** Seyoung Han, Taehyun Lee, Haeun Kim, Ji Hyun Um, Dongkyoung Lee

**Affiliations:** ^1^ Department of Future Convergence Engineering Kongju National University Cheonan Republic of Korea; ^2^ Department of Chemical Engineering Kongju National University Cheonan Republic of Korea; ^3^ Center for Future Sustainable Technology Kongju National University Cheonan Republic of Korea; ^4^ Global Institute of Manufacturing Technology (GITECH) Kongju National University Cheonan Republic of Korea; ^5^ Department of Mechanical and Automotive Engineering Kongju National University Cheonan Republic of Korea; ^6^ Department of Semiconductor Engineering Kongju National University Cheonan Republic of Korea

**Keywords:** aqueous zinc batteries, dendrite‐free Zn deposition, hole opening ratio, laser‐patterned Zn anode, nucleation density

## Abstract

Zinc metal is a promising anode for aqueous zinc batteries due to its high theoretical capacity and low cost. However, its practical implementation remains limited by dendrite formation and interfacial instability. Herein, we present a geometry‐driven strategy that regulates Zn deposition through control of the hole opening ratio (HOR) in laser‐ablated three‐dimensional hole‐patterned Zn foil. Based on preferential Zn deposition at periodically arranged hole edges, HOR was introduced as a geometric parameter that dictates the spatial density of nucleation sites. Systematic variation of the HOR reveals that it governs the dispersion and size of early‐stage Zn deposits, defining the electrode‐wide nucleation pattern and subsequent growth behavior. The optimized HOR balances sparse nucleation with localized growth and dense nucleation with coalescence‐driven aggregation, enabling spatially uniform Zn deposition. As a result, the HOR‐tailored electrode achieves dendrite‐free deposition with long‐term stability in symmetric cells (3400 h at 0.5 mA cm^−2^) and maintains stable cycling in full cells paired with a Zn pre‐intercalated NH_4_V_4_O_10_ cathode (1000 cycles at 2 A g^−1^). By establishing HOR‐controlled nucleation density as a geometry‐based design principle, this defect‐engineered and materials‐independent approach provides a general design framework for high‐performance aqueous metal batteries.

## Graphical abstract

Laser‐ablated 3D hole patterning enables geometry‐driven control of Zn deposition in aqueous batteries. The hole opening ratio (HOR) governs electrode‐wide nucleation patterns by regulating the spatial distribution of nucleation sites and the evolution of early‐stage Zn deposits. Optimized HOR balances localized growth and coalescence‐driven aggregation, resulting in hole‐directed dendrite‐free deposition and long‐term interfacial stability in aqueous zinc batteries.

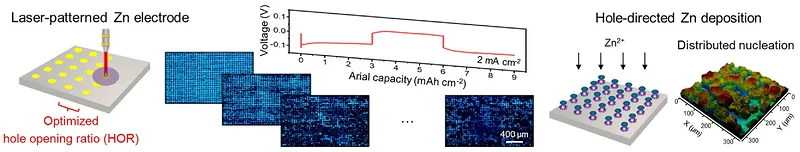

## 1 Introduction

The growing demand for advanced energy storage technologies has accelerated the development of diverse rechargeable battery technologies with improved safety and cost‐effectiveness [1]. Zinc‐based aqueous batteries are promising for sustainable energy storage due to their high theoretical capacities (820 mAh g^−1^ and 5855 mAh cm^−3^), low redox potential (−0.763 V vs. standard hydrogen electrode), natural abundance, and intrinsic safety enabled by aqueous electrolytes. However, various side reactions, including hydrogen evolution, corrosion, and Zn‐based byproduct formation, hinder the long‐term cycling stability of battery [2, 3, 4]. Notably, non‐uniform Zn deposition promotes dendritic growth during plating, resulting in uneven stripping and electrochemically inactive Zn, which exacerbates interfacial instability and shortens cycle life [5, 6, 7, 8]. Uniform and compact Zn deposition not only suppresses dendrite formation but also provides a basis for controlling the non‐uniform dissolution. This underscores the critical role of the deposition process and necessitates precise regulation of initial nucleation.

Commercially available Zn foil possesses a heterogeneous surface with defects such as vertical step edges, grain domains, and chemical impurities, resulting in uncontrolled Zn deposition. Endowing Zn foil with the ability to spatially and uniformly control Zn^2+^ ions has been identified as a key factor. The introduction of artificial seeds with strong affinity has emerged as an effective strategy for regulating Zn nucleation. A high density of zincophilic sites lowers the nucleation energy barrier and homogenizes the local current distribution, which in turn facilitates dendrite‐free plating and stripping. A wide range of metals [9, 10, 11], alloys [12, 13, 14], inorganic compounds [15, 16, 17, 18, 19], and polymers [20, 21, 22] with zincophilicity have been explored. However, their high affinity for hydrated Zn^2+^ can increase the risk of parasitic side reactions, particularly under highly reductive interfacial conditions. Meanwhile, a zincophobic layer has been constructed to guide lateral Zn deposition beneath the layer, while suppressing the side reactions [23, 24, 25]. Due to the weaker interaction of hydrated Zn^2+^ with the zincophobic layer than with metallic Zn, the Zn^2+^ ions preferentially migrate beneath the layer. As a result, lateral deposition occurs underneath the layer. This behavior prevents penetration through the interfacial layer and suppresses dendritic growth. Recently, a philic‐phobic gradient interfacial layer has been developed for lithium, sodium, and zinc electrodes to address the common issue of dendrite formation [26, 27, 28]. The artificial layers invariably comprise a philic bottom and a phobic top. The philic bottom strengthens the interactions with storage metal ions by lowering the nucleation energy barrier, which promotes homogeneous nucleation and controlled crystal growth. The phobic top directs Zn^2+^ ions to laterally deposit beneath the protective phobic layer, which restrains the vertical propagation of dendrites. The philic–phobic interfacial layer offers an effective strategy for achieving highly reversible metal anodes. However, its fabrication and integration remain complex, and the dual interfacial structure induces additional resistance at the electrode‐electrolyte interface.

In this study, we introduce three‐dimensional (3D) hole patterns into Zn foil as artificial defects, which act as distributed nucleation sites that direct spatially selective Zn deposition at the hole edges. To achieve precise dimensional control, laser‐based fabrication is employed, ensuring high reproducibility and structural fidelity. Without incorporating foreign materials, the patterned architecture preserves electrode integrity, facilitates mass transport, and mitigates side reactions. While affinity‐inducing species effectively promote Zn deposition, their strong affinity requires material‐specific verification of reversible dissolution in each cycle. In contrast, geometric electrode structuring is inherently free from this constraint. Building on the preferential deposition behavior at periodically arranged hole edges, the hole opening ratio (HOR) is introduced as a key geometric parameter that governs the spatial density of nucleation sites and regulates electrode‐wide deposition behavior. Consequently, the 3D hole‐structured electrode, without zincophilic and/or zincophobic materials, exhibits enhanced structural stability during prolonged cycling. Systematic HOR engineering reveals its role in balancing sparse and dense nucleation, enabling dendrite‐free Zn deposition with stable cycling performance in symmetric cells (3400 h at 0.5 mA cm^−2^). This behavior is further confirmed in full cells paired with a Zn pre‐intercalated NH_4_V_4_O_10_ cathode (1000 cycles at 2 A g^−1^), demonstrating the viability of HOR‐guided geometric design for stable aqueous metal batteries.

## 2 Results and Discussion

**FIGURE 1:**
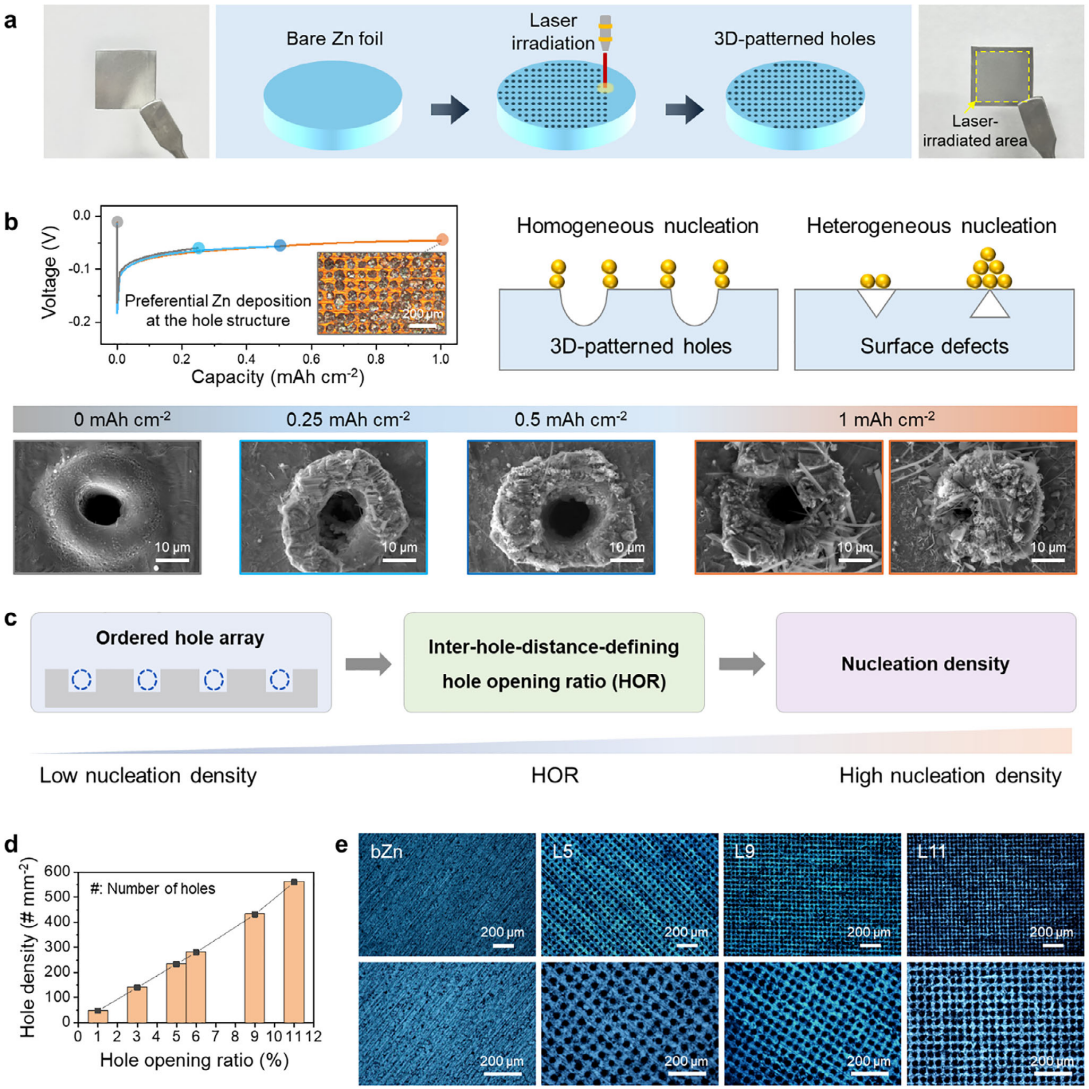
(a) Schematic illustration of laser ablation process for fabricating a 3D hole‐patterned architecture on Zn foil electrode. Photographs show the Zn foil before and after laser irradiation; (b) Time‐dependent evolution of Zn deposition at the patterned holes, highlighting the preferential nucleation followed by uniform Zn growth. Ex situ SEM images show the morphological evolution at high‐curvature hole edges during Zn plating (0, 15, 30, and 60 min) at 1 mA cm^−2^; (c) Schematic illustration of HOR‐controlled nucleation density; (d) Correlation between the HOR and hole density; (e) OM images of bZn and laser‐patterned electrodes (L5, L9, and L11) with different HOR.

3D‐patterned surface holes were fabricated on Zn foil via laser ablation, as illustrated in Figure 1a, where the initially shiny surface of commercially available Zn foil was converted into a matte surface after laser irradiation. In this study, the resulting laser‐ablated Zn foil was directly employed as an anode in the aqueous zinc battery without any additional treatment. In the 3D hole‐patterned Zn foil anode, preferential Zn deposition occurs at the hole, initiating at a protrusion along the hole edges and progressively growing outward from the site (Figure 1b). In contrast to heterogeneous Zn deposition across surface defects on bare Zn foil, spatially constrained and uniform Zn deposition occurs at the patterned holes. Motivated by the preferential Zn deposition at the patterned holes (Figure 1b), the HOR was introduced as a geometric parameter for controlling the spatial density of nucleation sites, as illustrated in Figure 1c. Because the inter‐hole distance decreases with increasing HOR, the density of available nucleation sites can be systematically varied, enabling investigation of how nucleation density influences subsequent Zn growth behavior.

**FIGURE 2:**
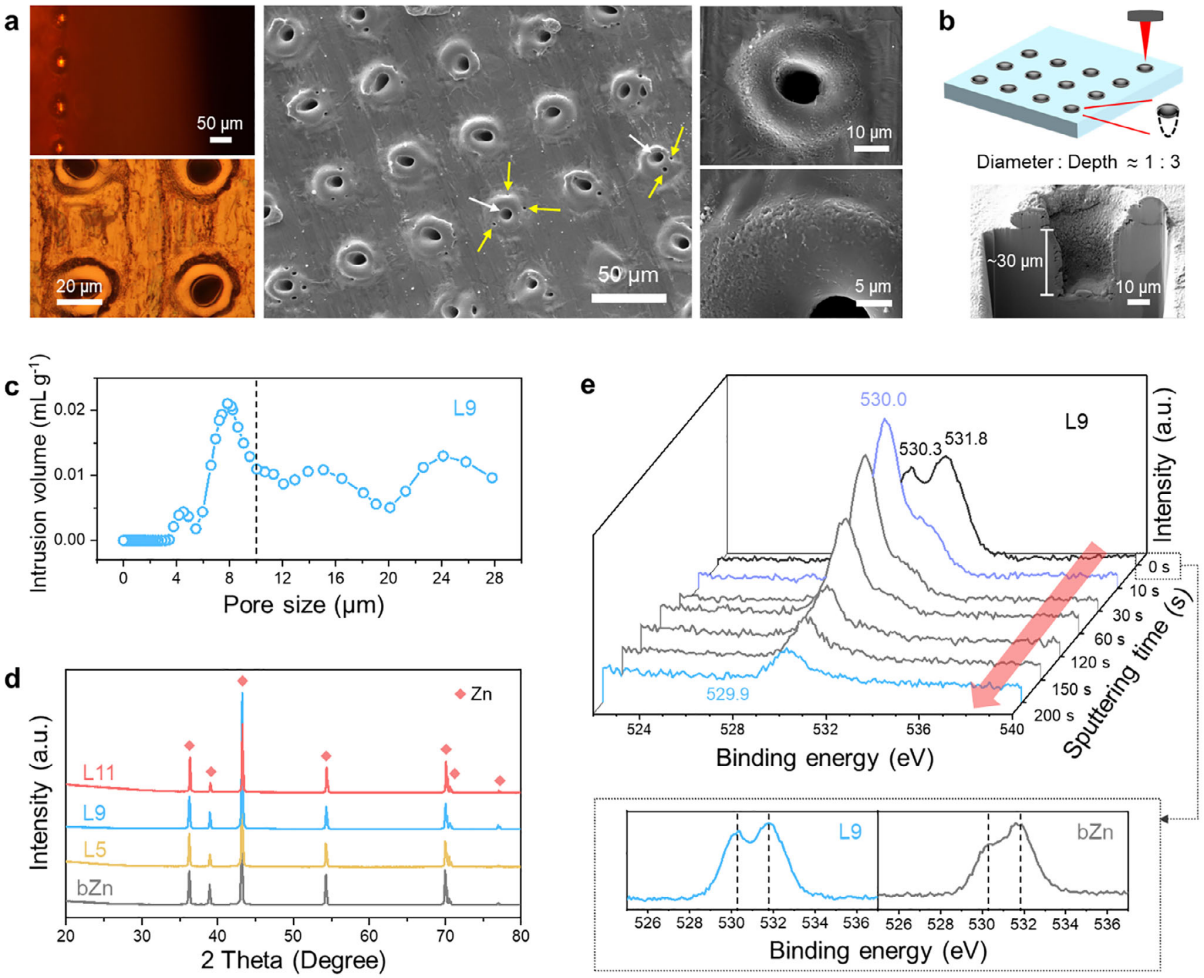
(a) OM and SEM images of the laser‐ablated Zn foil surface, showing the formation of periodically arranged holes; (b) Cross‐sectional FIB‐SEM image of a representative hole, showing its 3D geometry and depth; (c) Mercury intrusion porosimetry showing the pore size distribution of the laser‐ablated hole structures; (d) XRD patterns of laser‐patterned Zn and bZn electrodes; (e) XPS O 1s depth profiles of L9 electrode during Ar^+^ sputtering, with a comparison between L9 and bZn at the pristine state.

As shown in Figure 1d and Figure S1, the hole density varies systematically with laser irradiation conditions. The *x*‐axis presents the hole opening ratio (HOR), defined in the Experimental Section as the percentage of surface area occupied by holes. When the ratio exceeded 11%, the holes began to overlap with adjacent ones, thereby losing their independent structure. Accordingly, conditions exceeding 11% were excluded from the structural design. Electrodes with HOR of 5%, 9%, and 11% are hereafter referred to as L5, L9, and L11, respectively, while bare Zn foil is referred to as bZn. The hole density and size of L5, L9, and L11 were examined by optical microscopy (OM) and compared with those of bZn in Figure 1e. While bZn exhibits irregular surface defects, the laser‐ablated electrodes display uniformly arranged surface holes with well‐defined patterned morphology. As HOR increases, the hole density increases, leading to a reduction in the inter‐hole distance. Additional texture variations induced by laser processing are observed along the hole edges, as shown in Figure S2, and will be further discussed in Figure 2a. The increase in hole density observed by imaging analyses is supported by contact angle measurements (Figure S3). Higher hole density facilitates electrolyte infiltration into the holes, consistent with increased internal void volume within the electrode. Figure 2a presents magnified surface morphologies of the laser‐ablated electrode, where aligned holes with a bright, flash‐like appearance are observed in the OM image. Compared with Figure 1e, higher‐magnification imaging (Figures S2 and S4) shows burr‐like protrusions along the hole edges associated with laser ablation. Secondary holes (indicated by yellow arrows) adjacent to the primary hole (indicated by a white arrow) are observed. These features are attributed to instantaneous melting and re‐solidification driven by spatially non‐uniform energy deposition and localized heat accumulation. This process results in the formation of a heat‐affected zone [29, 30]. Additionally, scanning electron microscopy (SEM) images in Figure 2a show a ∼10 µm‐diameter hole with a micro‐nodular surface texture, arising from the laser‐induced localized thermal effects. Cross‐sectional focused ion beam‐scanning electron microscopy (FIB‐SEM) imaging presents a hole depth of ∼30 µm as shown in Figure 2b. The entrance diameter‐to‐depth ratio of the 3D‐patterned hole is approximately 1:3. The laser‐fabricated hole exhibits a slight tapering along the depth direction while maintaining a well‐defined bottom surface. Mercury porosimetry analysis (Figure 2c) reveals that the diameters of patterned holes range from ∼3 to 28 µm, indicating the presence of secondary holes and a broad distribution of hole sizes.

All laser‐ablated electrodes exhibit x‐ray diffraction (XRD) patterns identical to bZn, corresponding to metallic Zn (PDF#04‐0831), confirming that their metallic character is preserved after laser processing (Figure 2d). To further investigate the chemical states of electrode after laser ablation, x‐ray photoelectron spectroscopy (XPS) depth profiling was performed by argon ion sputtering (Figure 2e and Figure S5). In the L5, L9, and L11 series, L9 electrode, which has an intermediate HOR, was selected to compared with bZn. The survey spectra of L9 and bZn (Figure S5a) confirm that no additional chemical species are formed after laser processing. The O 1s spectra of both L9 and bZn exhibit two distinct peaks (Figure 2e and Figure S5b), attributed to the strong oxygen affinity of Zn and the formation of a native oxide layer [31, 32]. At a sputtering time of 0 s, two O 1s peaks corresponding to ZnO (∼530 eV) and Zn(OH)_2_ (∼532 eV) are observed in both L9 and bZn, confirming the presence of surface oxide and hydroxide species. Concurrently, Figure S5c shows the Zn 2p spectra, indicating the coexistence of Zn^0^ and Zn^2+^ species due to partial surface oxidation. Compared with bZn, L9 exhibits a higher fraction of ZnO, which is associated with laser‐induced surface oxidation in addition to the native oxide layer. Nevertheless, the low oxygen content, which is below the detection limit of XRD, reveals that the outermost surface layer of both L9 and bZn consists predominantly of Zn^0^, with a minor contribution from Zn^2+^ species. Furthermore, as sputtering progresses into the electrode interior, oxidation‐related signals associated with native and laser‐induced oxide species gradually decrease in L9, confirming that laser processing does not substantially alter the overall oxidation state of Zn [31, 33].

**FIGURE 3:**
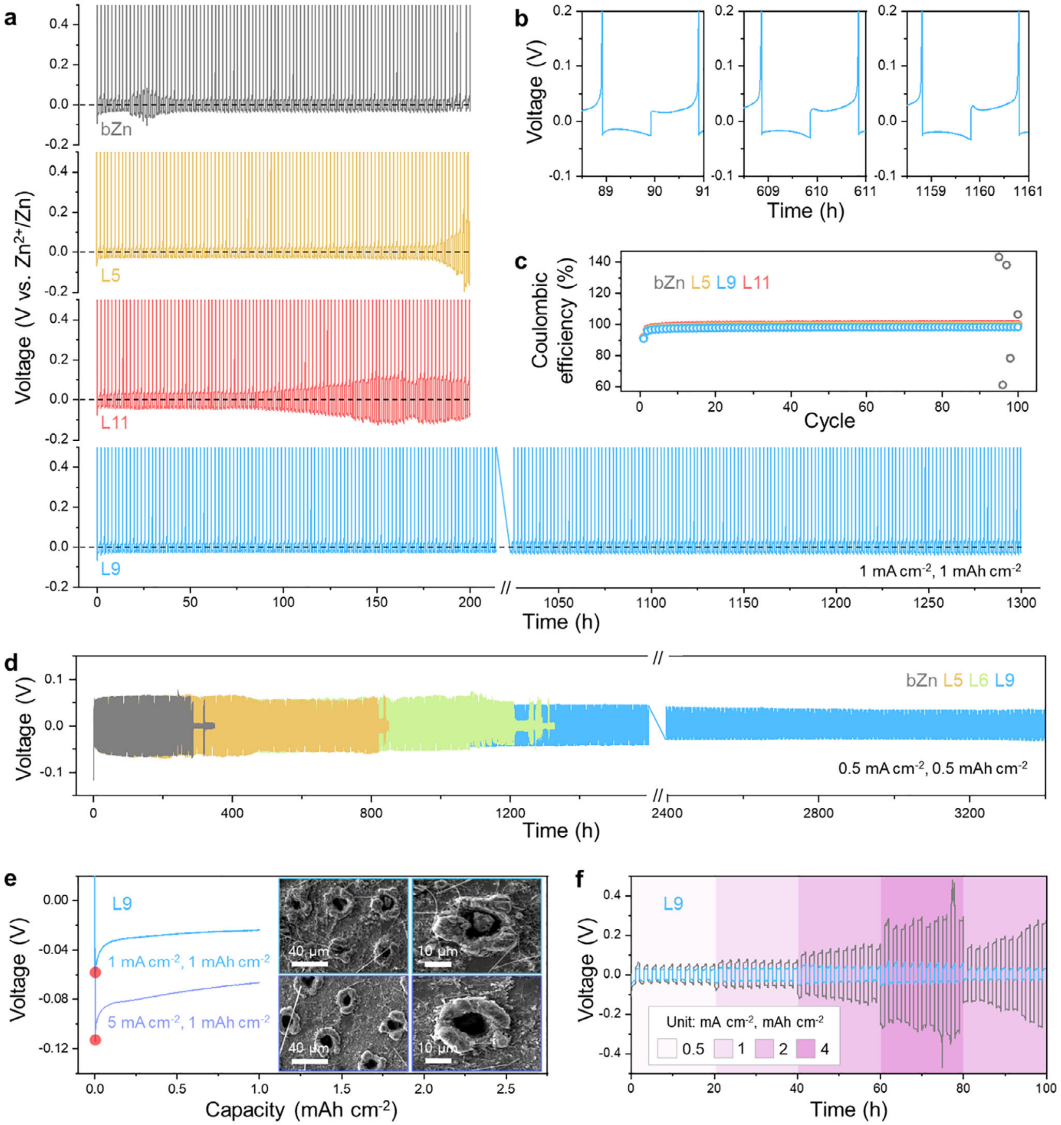
(a) Time‐voltage profiles of laser‐patterned Zn and bZn electrodes in asymmetric Zn||Cu cells at 1 mA cm^−2^ with 1 mAh cm^−2^; (b) Representative time‐voltage profiles of L9||Cu cell after 89, 609, and 1159 h of operation; (c) CE of laser‐patterned Zn and bZn electrodes in Zn||Cu cells over 100 cycles; (d) Cycling performance of laser‐patterned Zn symmetric cells at 0.5 mA cm^−2^ with 0.5 mAh cm^−2^; (e) Capacity‐voltage profiles of L9 electrode at different current densities under a fixed capacity of 1 mAh cm^−2^. Insets show SEM images of Zn deposition at different current densities, presented at different magnifications; (f) Rate performance of L9 and bZn symmetric cells over a range of current densities (0.25–4 mA cm^−2^) and areal capacities (0.25–4 mAh cm^−2^).

Electrochemical behavior of the 3D hole‐patterned electrodes was evaluated in Figure 3. Figure 3a presents the time‐voltage profiles of Zn plating/stripping in an asymmetric Zn||Cu cell, where copper foil serves as a counter electrode. Zn is deposited at a current density of 1 mA cm^−2^ for 1 h and stripped to a cut‐off voltage of 0.5 V. bZn undergoes intermittent increases in nucleation overpotential after the initial ∼20 h, followed by failure at ∼190 h. Compared to bZn, the laser‐fabricated electrodes exhibit more stable Zn plating/stripping behavior. Among the L5, L9, and L11 electrodes, the intermediate configuration L9 presents the longest cycling stability over 1300 h. L5 shows cycling stability comparable to that of L9 for ∼180 h, after which the overpotential increases sharply. L11 shows a higher nucleation overpotential than L5 and L9 even in the initial cycles, and this overpotential further increases during continued cycling. Figure S6 compares the first nucleation overpotentials of all the electrodes. While bZn presents the highest nucleation overpotential, the 3D hole‐patterned electrodes exhibit lower values, consistent with improved cycling stability [34, 35, 36]. As shown in Figure 3b, L9 presents no noticeable increase in overpotential at ∼89, 609, and 1159 h over 1300 h of operation. Coulombic efficiencies (CEs) of L9 remain close to 100% for 1300 h, confirming the stable electrochemical operation enabled by the patterned structure (Figure 3c and Figure S7). Upon increasing the areal capacity from 1 to 2 mAh cm^−2^, L9 maintains stable plating/stripping behavior, as shown in Figure S8. Even at the higher deposition capacity, the negligible change in overpotential is consistent with sustained interfacial stability during cycling. Cyclic voltammetry (CV) was performed using the asymmetric cell configuration in Figure S9. All the electrodes exhibit similar CV profiles and peak positions, indicating the absence of additional electrochemical reactions. Furthermore, Tafel polarization measurement (Figure S10) reveals comparable corrosion current densities among the bare Zn and laser‐patterned electrodes, indicating that the laser‐patterned geometry does not substantially alter the intrinsic corrosion behavior. A recent study reported that a functional surface coating can improve corrosion resistance by suppressing water‐induced side reactions [37]. In contrast, these results indicate that the differences between the bare Zn and laser‐patterned electrodes are unlikely to originate primarily from variations in corrosion behavior.

**FIGURE 4:**
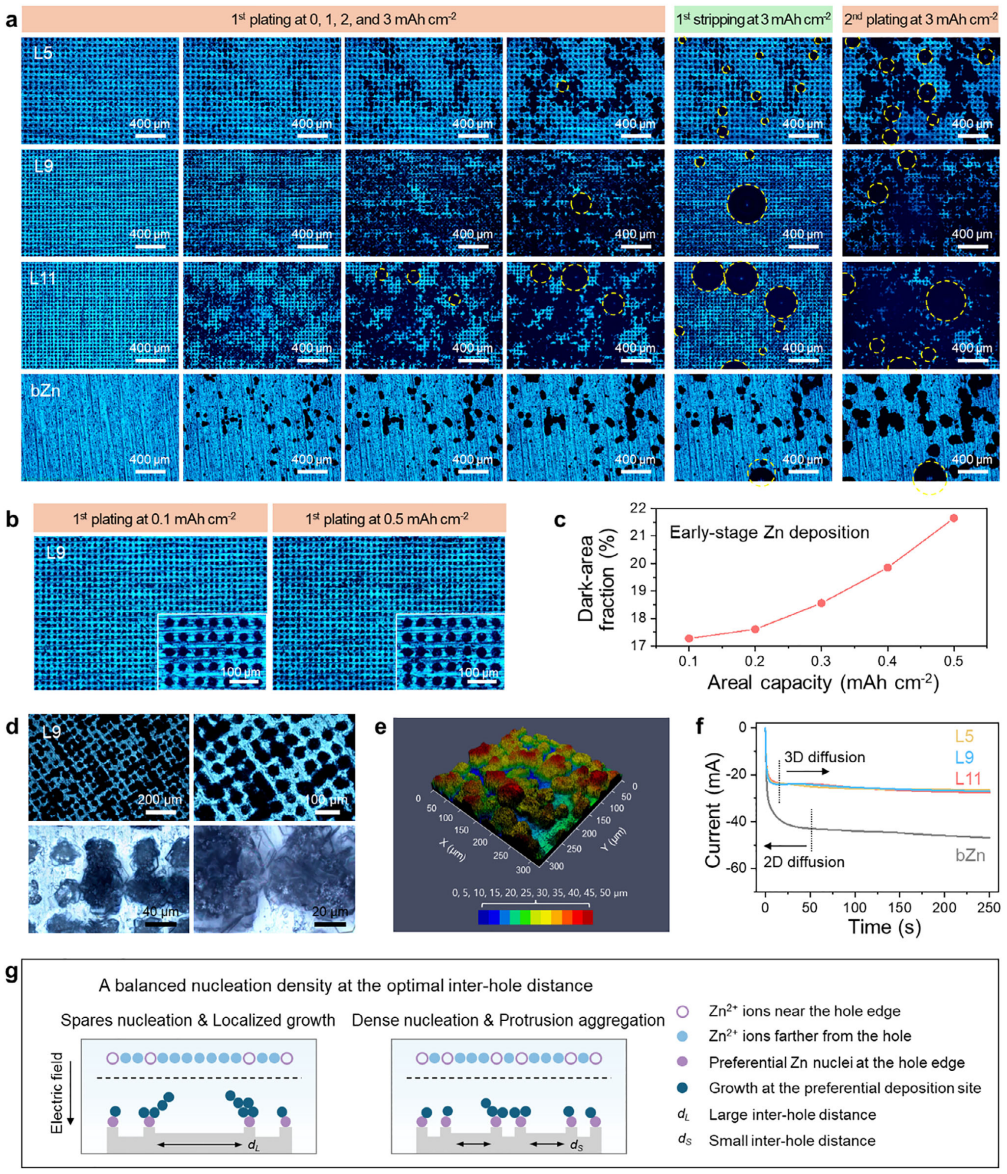
a) Operando OM images of laser‐patterned Zn and bZn electrodes during the 1st Zn plating (0, 1, 2, and 3 mAh cm^−2^), 1st stripping at 3 mAh cm^−2^, and 2nd plating to 3 mAh cm^−2^ at a current density of 2 mA cm^−2^; (b) Operando OM images of L9 at 0.1 and 0.5 mAh cm^−2^ during the early stage of Zn deposition, with enlarged insets; (c) Quantitative analysis of the thresholded dark‐area fraction as a function of areal capacity during the early stage of Zn deposition; (d) OM images of L9 electrode plated to 1.5 mAh cm^−2^ at 1 mA cm^−2^; (e) LSCM image and corresponding height map of L9 electrode after Zn plating at 1 mA cm^−2^ with 1 mAh cm^−2^; (f) CA curves of laser‐patterned Zn and bZn electrodes; (g) Schematic illustration of nucleation density as a function of the HOR. The optimized hole geometry balances sparse nucleation with localized growth and dense nucleation with protrusion aggregation, enabling uniform Zn deposition.

Galvanostatic plating/stripping was evaluated in a symmetric cell configuration at a current density of 0.5 mA cm^−2^ with a capacity of 0.5 mAh cm^−2^ (Figure 3d). Because L5, which has a lower HOR than L11, exhibited enhanced cycling stability (Figure 3a), L5 was further evaluated electrochemically together with L9. Additionally, an intermediate HOR (6%) was introduced as a representative condition between L5 and L9 for systematic comparison. Consistent with the cyclability trend observed in the asymmetric cell (Figure 3a), it is evident that increasing the HOR from 5 to 6% further extends the cycle life. L9 maintains stable cycling over 3400 h, indicating that the 3D‐patterned hole structure with an optimized HOR promotes uniform and reversible Zn plating/stripping. To further examine the long‐term cycling behavior of the L9 electrode, locally magnified voltage profiles recorded after 500, 1000, 2000, and 3000 h of cycling are provided in Figure S11. Although the slightly higher ZnO fraction on L9, observed in Figure 2e, may contribute to Zn nucleation to a limited extent due to its zincophilic nature, ZnO and Zn(OH)_2_ species are also detected on bZn as a result of native oxidation. Considering the significantly improved electrochemical performance of L9, this improvement is predominantly attributed to the laser‐patterned geometry, although a minor contribution from the slightly increased surface ZnO content cannot be entirely excluded. A systematic comparison with recently reported 3D‐structured Zn anodes is provided in Table S1, highlighting the competitive electrochemical performance of the laser‐patterned Zn anode. To further assess the electrochemical behavior of the laser‐patterned Zn anode under higher current‐density and areal‐capacity conditions, additional Zn||Zn symmetric‐cell cycling was performed at a current density of 5 mA cm^−2^ with a capacity of 10 mAh cm^−2^ (Figure S12. High depth of discharge (DOD) has been recognized as an important metric for evaluating the practical utilization of Zn metal anodes, reflecting the fraction of Zn actively involved in repeated plating/stripping processes [38]. Based on the actual Zn mass remaining after laser ablation, the applied areal capacity corresponds to a DOD of approximately 36.4% for the L9 electrode. The L9 electrode maintains a low and stable voltage polarization throughout 100 h of cycling, whereas bZn exhibits substantially increased polarization and pronounced voltage fluctuations. These results demonstrate that the laser‐patterned L9 electrode sustains stable and reversible Zn plating/stripping under high current density and high areal‐capacity condition, further supporting its practical applicability. Figure 3e and Figure S13 show the capacity‐voltage profiles of L9 and bZn at different current densities of 1 and 5 mA cm^−2^ under a fixed deposition capacity of 1 mAh cm^−2^, with the corresponding top‐view SEM images. At the lower current density, both L9 and bZn exhibit localized and spatially heterogeneous Zn deposition after plating. In the L9 electrode, Zn deposits are predominantly observed on protrusions located along the hole edge, while a particle is also observed within the hole cavity. Due to the post‐plating nature of the observation, the particle cannot be unequivocally identified as a primary nucleation site. Instead, the post‐plating morphology reflects the cumulative growth of Zn deposits rather than the initial nucleation location itself (Figure 1b). Therefore, the particle observed within the hole cavity may represent a secondary deposit formed during the continued growth of protrusion‐initiated deposits, consistent with diffusion‐limited deposition behavior. In contrast, increasing the current density promotes more spatially uniform Zn deposition across the electrode surface. This interpretation is further corroborated by the subsequent operando observations and quantitative analysis presented in Figure 4. Rate performance of L9 and bZn was also evaluated in the symmetric cell (Figure 3f). The current density increases from 0.5 to 4 mA cm^−2^ while maintaining a fixed plating/stripping time of 1 h. Compared with bZn, L9 exhibits consistently lower polarization across all current densities and retains stable cycling behavior after exposure to 4 mA cm^−2^, demonstrating highly reversible Zn plating/stripping and robust structural stability. To further evaluate Zn plating/stripping kinetics, the apparent exchange current density (*i*_0_) was estimated by linearly fitting the relationship between the total voltage hysteresis and the applied current density obtained from the Zn||Zn symmetric‐cell rate measurement (Figure 3f) [39]. As shown in Figure S14, the L9 electrode exhibits a higher apparent *i*_0_ than bZn, indicating more favorable Zn plating/stripping kinetics under the investigated current‐density range. Along with the lower Zn nucleation overpotential (Figure S6), these results suggest that the laser‐patterned geometry lowers the effective nucleation barrier and reduces polarization during Zn plating/stripping, improving the overall Zn plating/stripping kinetics.

Ex situ SEM analysis was conducted at different stages of Zn deposition as shown in Figure 1b and Figure S15. Zn deposition initiates at the surface protrusions and progressively thickens, indicating spatially coordinated nucleation guided by the 3D hole architecture. The high‐curvature regions are expected to amplify the local electric field and localize current density, promoting preferential Zn deposition at the hole edges, while the periodic hole arrangement facilitates spatially uniform Zn deposition across the electrode. Operando OM imaging (Figure S16 and Movie S1) further confirms this preferential and uniform Zn deposition at the hole structure. Zn deposition, dissolution, and subsequent redeposition of all the electrodes were examined by operando OM at a current density of 2 mA cm^−2^ with a capacity of 3 mAh cm^−2^ (Figure 4a and Figures S17–S20 and Movies S2‐S5). As shown in Figure S16 and Movie S1, Zn deposition at 1 mAh cm^−2^ produced insufficient morphological contrast to clearly visualize the spatial evolution of deposition across the electrode. Therefore, operando visualization was conducted at 3 mAh cm^−2^. In Figure 4a, gas bubbles generated during plating/stripping appear as symmetric circular features, highlighted by yellow dashed circles. It is highly probable that additional bubbles, which were difficult to distinguish from Zn deposits in the OM imaging, were also present within the field of view. bZn exhibits sparse nucleation followed by progressive growth during plating, ultimately leading to dendrites reaching ∼100 µm in size. Upon stripping, the large deposits undergo incomplete dissolution and act as preferential nucleation sites in subsequent cycles, thereby leading to the formation of larger dendrites during redeposition. This result underscores the critical role of uniform and compact deposition in achieving high reversibility. In contrast, all the 3D‐patterned electrodes suppress localized Zn growth and promote spatially distributed deposition. As the first plating proceeds up to a capacity of 3 mAh cm^−2^, the spatial distribution of Zn deposits varies with the HOR. While L9 maintains electrode‐wide uniform Zn deposition, L5 and L11 exhibit progressive growth. A substantial portion of this growth occurs at geometric sites similar to those observed at a capacity of 1 mAh cm^−2^, indicating growth‐dominated deposition (Figure 4a and Figure S21).

Moreover, compared to bZn, all the 3D‐patterned electrodes show clearly distinguishable dissolution behavior during stripping. Among L5, L9, and L11, residual deposits are observed exclusively on the L5 electrode after stripping at 3 mAh cm^−2^, where they subsequently act as preferential nucleation sites during subsequent plating (Figure 4a and Figure S22). This observation implies that the dispersion and corresponding size of the initial deposits are determined by the HOR, which in turn continuously affects the subsequent dissolution and redeposition processes. To further examine the early‐stage deposition behavior, operando OM images of the L9 were analyzed at areal capacities ranging from 0.1 to 0.5 mAh cm^−2^ (Figure 4b). The laser‐fabricated holes, which appear as initially dark regions as observed in Figure 4a, gradually expand with increasing capacity during Zn plating (Movie S3). This progressive expansion suggests that Zn growth occurred over a broad distribution of hole sites rather than being concentrated at only a few localized locations. To quantitatively evaluate this behavior, the thresholded dark‐area fraction was determined under identical analysis conditions. As shown in Figure 4c, the thresholded dark‐area fraction gradually increases with increasing capacity, indicating the progressive expansion of the deposition area during the initial stage of Zn deposition. These observations provide further experimental support for the mechanism that 3D electrode geometry can modify the spatial distributions of the local electric field, Zn^2+^ ion flux, and current density to regulate Zn deposition [40]. Based on these observations, the Zn deposition behavior can be interpreted as follows. The high‐curvature hole edges serve as geometrically favorable Zn nucleation sites through local electric‐field enhancement. However, the resulting deposition geometry fundamentally differs from the classical tip‐effect scenario. Rather than concentrating the local electric field at only a few isolated locations, the periodically distributed hole array provides multiple geometrically favorable nucleation sites across the electrode surface, enabling simultaneous Zn nucleation and subsequent expansion through the concurrent growth of these spatially distributed nuclei. As a result, electrode‐wide uniform deposition arises not from the disappearance of edge‐preferred deposition, but from the concurrent growth of numerous spatially distributed preferential deposition sites. Figure 4d and Figure S23 show that Zn deposits progressively overgrow the holes, with some evolving into interconnected structures via protrusive growth that establishes contact with adjacent deposits. In contrast to the localized dendritic growth observed on bZn, the alignment of these 3D Zn protrusions with the hole‐patterned architecture is further confirmed by laser scanning confocal microscopy (LSCM) in Figure 4e and Figure S24. In Figure 4f, the chronoamperometry (CA) curve of bZn shows a continuously decreasing current density over 50 s, indicative of diffusion‐controlled growth with limited nucleation density. By comparison, the 3D‐patterned electrodes show a more rapid current stabilization at lower current densities, suggesting that the patterned structure facilitates more rapid nucleation and uniform Zn growth [34, 41]. Combined with the higher apparent *i*_0_ (Figure S14), the faster stabilization of the current response to a lower steady‐state current further supports the interpretation that the laser‐patterned geometry promotes more favorable Zn plating/stripping kinetics.

Based on the above observations, the schematic in Figure 4g illustrates how HOR determines the nucleation density and subsequent Zn growth behavior. In L5, a large inter‐hole distance (*d_L_*) leads to sparse nucleation and localized growth, whereas L11, with a small inter‐hole distance (*d_S_*), promotes dense nucleation and protrusion aggregation through coalescence of neighboring nuclei. Meanwhile, the optimized L9 structure achieves a balanced nucleation and growth behavior, enabling a high nucleation density with suppressed agglomeration. As a result, the 3D hole‐patterned architecture with optimized HOR effectively controls the spatial distribution of nucleation and modulates the subsequent dissolution and redeposition processes. To further investigate how the HOR‐dependent deposition behaviors influence the subsequent Zn morphology evolution during cycling, post‐cycling OM and SEM observations were performed (Figure S25). After 25 cycles, the L5 electrode exhibits large spatially localized Zn deposits, suggesting that the preferentially formed deposits during the initial deposition stage continued to undergo localized growth at a limited number of deposition sites. In contrast, the L11 electrode develops extensive deposition regions, consistent with continued coalescence among neighboring deposits resulting from the relatively small inter‐hole spacing. Meanwhile, the L9 electrode maintains comparatively homogeneous electrode‐wide deposition even after 50 cycles, indicating that the balanced nucleation and growth behavior enabled by the optimized HOR was largely preserved throughout cycling. These observations suggest that the HOR‐dependent deposition behavior established during the initial deposition stage largely determines the subsequent morphology evolution of the laser‐patterned electrodes. Meanwhile, to further evaluate whether the laser‐patterned architecture remained structurally intact after repeated Zn plating/stripping, cross‐sectional FIB‐SEM observations were performed (Figure S26). The results confirm that the optimized L9 electrode maintains its original laser‐patterned hole architecture without obvious structural collapse after repeated Zn plating/stripping, indicating that the hole structure remained mechanically intact. Additionally, post‐cycling XRD analysis was performed on the bZn and L9 electrodes after 50 cycles to examine the formation of side‐reaction‐derived byproducts (Figure S27). The diffraction peak assignable to Zn_4_SO_4_(OH)_6_∙xH_2_O is absent in both pristine electrodes but becomes observable after 50 cycles for both the bZn and L9 electrodes. These observations indicate that comparable Zn‐based byproduct formation occurred on both electrodes during cycling.

**FIGURE 5:**
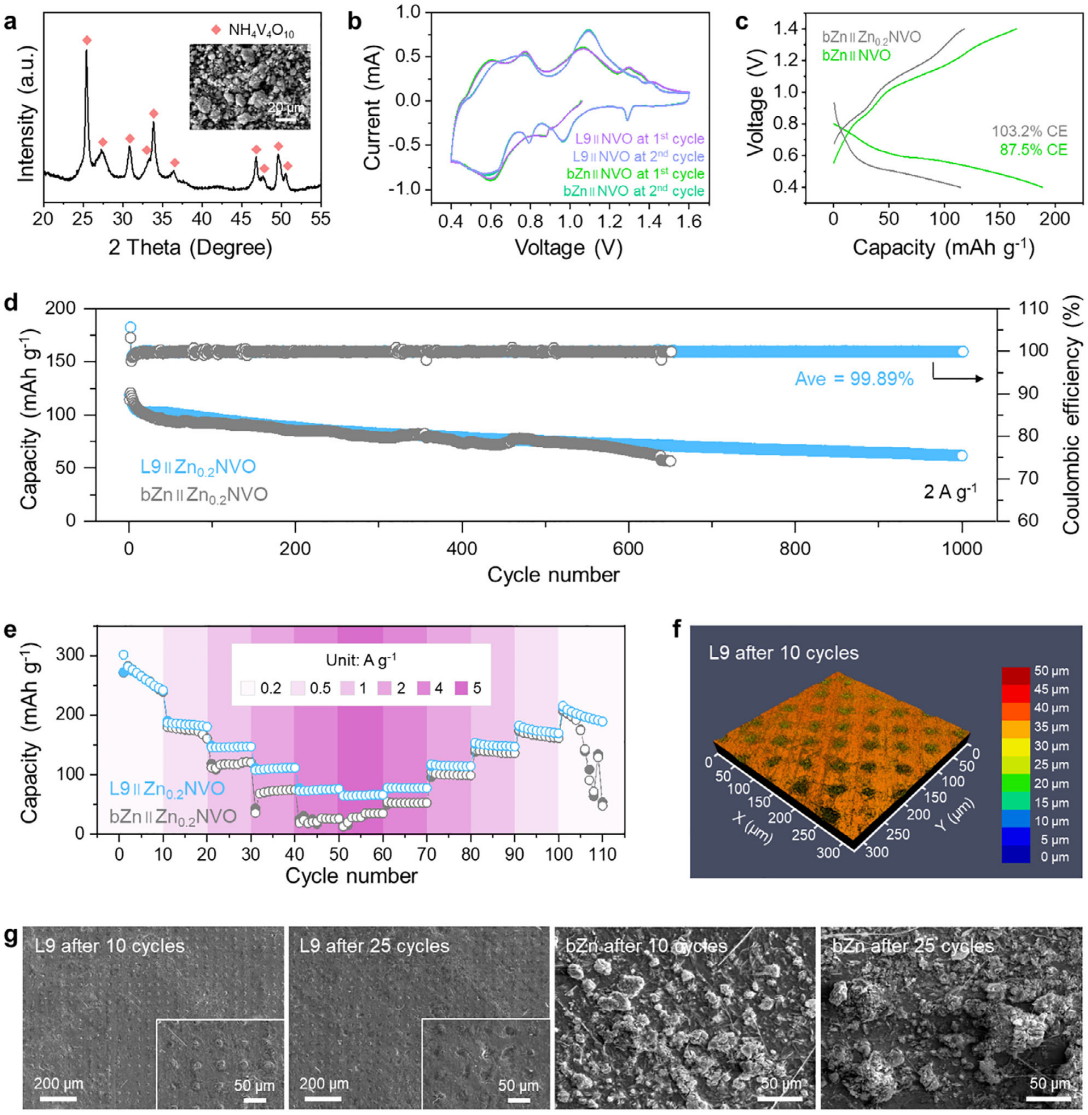
(a) XRD pattern of the synthesized NH_4_V_4_O_10_ powder with an inset SEM image; (b) CV curves of L9||NVO and bZn||NVO full cells for the initial two cycles; (c) Initial galvanostatic charge–discharge profiles of bZn||NVO and bZn||Zn_0.2_NVO, where bZn||Zn_0.2_NVO corresponds to the first cycle following the pre‐intercalation process; (d) Cycling performance and corresponding CE of L9||Zn_0.2_NVO and bZn||Zn_0.2_NVO full cells at 2 A g^−1^; (e) Rate capability of L9||Zn_0.2_NVO and bZn||Zn_0.2_NVO at various current densities (0.2–5 A g^−1^); (f) LSCM image and corresponding height map of L9 anode after 10 cycles at 2 A g^−1^; (g) SEM images of L9 and bZn anodes after 10 and 25 cycles at 1 A g^−1^.

The spatially coordinated and uniform Zn deposition enabled by 3D hole patterning was further validated in a full cell configured with an NH_4_V_4_O_10_ cathode (hereafter referred to as NVO). The prepared NVO exhibits a layered NH_4_V_4_O_10_ crystal structure (PDF#31‐0075) and an agglomerated granular morphology composed of irregular particles with a broad size distribution, as shown in Figure 5a. In the CV curves of L9||NVO and bZn||NVO full cells (Figure 5b and Figure S28), multistep redox behavior arising from the multivalent vanadium chemistry, coupled with Zn^2+^ intercalation and de‐intercalation, is observed. The redox peaks at ∼1.29/1.41 V from the second cycle onward are indicative of reversible NH_4_^+^ intercalation/de‐intercalation, confirming the participation of NH_4_^+^ in the electrochemical reactions [42, 43, 44]. Considering that pre‐intercalated cations mitigate structural instability in layered materials by reinforcing the interlayer framework [45, 46, 47], Zn^2+^ was electrochemically pre‐intercalated into NVO prior to its use as a cathode in this study. As shown in Figure S29, the extent of Zn^2+^ pre‐intercalation was controlled by applying a calculated charge according to the mass loading and desired Zn stoichiometry (0.1, 0.2, and 0.5 Zn per formula unit), producing Zn_0.1_NVO, Zn_0.2_NVO, and Zn_0.5_NVO. Figure 5c shows the first capacity‐voltage profiles of bZn||Zn_0.2_NVO and bZn||NVO. The reduced discharge capacity of bZn||Zn_0.2_NVO is attributed to the partial occupation of Zn^2+^ storage sites, which restricts further Zn^2+^ insertion. The CE exceeding 100% indicates partial extraction of the pre‐intercalated Zn^2+^ during charging, resulting in a charge capacity that surpasses the corresponding discharge capacity. The cycling behavior (Figure S30) confirms that Zn^2+^ pre‐intercalation enhances the structural stability of NVO. Furthermore, among the pre‐intercalated compositions, Zn_0.2_NVO is identified as the optimal condition based on its superior cycling stability (Figure S31). Compared to bZn||Zn_0.2_NVO, L9||Zn_0.2_NVO full cell demonstrates stable long‐term cycling performance over 1000 cycles at 2 A g^−1^ (Figure 5d). The L9||Zn_0.2_NVO full cell also exhibits excellent rate capability over a wide range of current densities, along with a good capacity recovery after high‐rate cycling (Figure 5e and Figure S32). To further evaluate the high‐rate full‐cell performance, additional cycling results at 3 A g^−1^ are provided in Figure S33.

Figure 5f shows LSCM image of L9 anode after 10 cycles at a current density of 2 A g^−1^. The L9 electrode maintains its patterned architecture and displays a smooth and spatially uniform surface without high‐aspect‐ratio dendritic features, indicative of stabilized Zn plating. Interestingly, compared with the initially plated surface of L9 in Figure 4e, the 3D Zn protrusions aligned with the hole‐patterned structure become noticeably smoother upon repeated cycling, with the development of areal Zn deposition in the inter‐hole regions. This morphological evolution is likely associated with the gradual redistribution of local current density among the periodically arranged protrusions during repeated plating/stripping. This process leads to increasingly uniform Zn deposition in the inter‐hole regions. This behavior is consistent with previous reports on structured Zn electrodes, where engineered microstructures homogenize the electric field and ion flux, thereby promoting uniform Zn growth during cycling [40, 48]. Additionally, SEM imaging after 10 cycles reveals pronounced dendrite formation on bZn, whereas L9 remains dendrite‐free, as shown in Figure 5g and Figure S34. After 25 cycles, severe dendritic growth is observed on bZn, consistent with progressive morphological instability. Conversely, L9 continues to exhibit stable and relatively smooth Zn deposition without noticeable dendritic formation, supporting sustained structural stability during extended cycling. The Zn deposition in the inter‐hole regions (Figure 5f) remains evident in the SEM images and further expands with continued cycling. This behavior suggests that the periodically arranged hole architecture promotes redistribution of local current density during cycling, enabling spatially uniform Zn deposition across the electrode surface.

## 3 Conclusion

In this study, we develop a laser‐ablated 3D hole‐patterned Zn anode as a geometry‐driven interfacial strategy for regulating spatial Zn deposition. Based on the preferential Zn deposition at periodically arranged hole edges, the HOR was introduced as a geometric design parameter for controlling the spatial nucleation density. Systematic variation of the HOR demonstrates that it governs the dispersion and size of early‐stage deposits, as well as subsequent dissolution and redeposition behavior, defining the electrode‐wide nucleation pattern and growth evolution. The optimized HOR balances the trade‐off between sparse nucleation, which promotes localized growth, and dense nucleation, which accelerates coalescence‐driven aggregation, enabling spatially uniform Zn deposition. Consequently, the optimized hole‐patterned Zn anode exhibits dendrite‐free performance in symmetric cells (3400 h at 0.5 mA cm^−2^) and maintains stable cycling in full cells paired with a Zn pre‐intercalated NH_4_V_4_O_10_ cathode (1000 cycles at 2 A g^−1^). Through geometry‐driven control of Zn deposition, this defect‐engineered and materials‐independent strategy preserves electrode integrity and supports long‐term stability in aqueous metal batteries. These findings establish the HOR‐controlled nucleation density as a geometry‐based design principle and demonstrate the effectiveness of architectural engineering in directing metal deposition toward stable and uniform growth.

## 4 Experimental Section

### 4.1 Laser Structuring Process

In this study, a Zn foil (99.95%, MTI Co. Ltd.) with a thickness of 50 µm was used. The laser system employed is illustrated in Figure S35, operating at a wavelength of 1064 nm with a spot size of 30 µm and a working distance of 189 µm. A ytterbium nanosecond pulsed fiber laser (IPG‐YLPM, IPG Photonics, USA) was utilized. The laser parameters are summarized in Table S1, where the power was varied from 6 to 20 W for the experiments. The pulse duration, repetition rate, and number of pulses were set to 4 ns, 500 kHz, and 200, respectively. Laser structuring was conducted over an area of 20 × 20 mm, and the percentage of the total area occupied by holes (A_opening_) was calculated using Equation (1). Here, A_hole_ represents the area occupied by the holes, and A_original_ denotes the area of the original electrode.

(1) $$A_{\mathrm{opening}}\left(\%\right)=\left(A_{\mathrm{hole}}\right)\;/\;\left(A_{\mathrm{original}}\right)\;x\;100$$

The hole distance between the generated holes is identical in both the vertical and horizontal directions.

### 4.2 Laser Parameter Study

The hole width generated at varying laser powers is shown in Figure S36. As the laser power increased, the hole width also increased. At powers exceeding 16 W, the high intensity caused damage to the Zn foil surface, resulting in through‐holes and specimen deformation (Region C). In the range of 8–16 W, a proper 3D structure was formed (Region B), while below 8 W, the power was insufficient to create a 3D structure (Region A). Additionally, as shown in Figure S37, representative photographs and OM images of Zn foils processed at 6 and 12 W confirmed the rationale for selecting 8 W by showing that insufficient laser power did not produce well‐defined 3D holes, whereas excessive laser power caused pronounced front‐side surface roughening and heat‐induced deformation on the back side of the thin Zn foil. Accordingly, a laser power of 8 W was selected for subsequent experiments.

### 4.3 Characterization Methods

Surface morphology of the electrodes was characterized by OM (LV150N, Nikon, Japan) and FE‐SEM (MIRA LMH, TESCAN, Czech Republic). Surface‐developed porosity of the electrodes was characterized by mercury intrusion porosimetry (AutoPore 9520, Micromeritics, USA). XRD (MiniFlex600, Rigaku, Japan) was performed using Cu Kα radiation to identify the crystal structure of the electrodes. Surface composition and chemical states were characterized by XPS (K‐Alpha, Thermo Fisher Scientific, USA). XPS depth profiling was conducted using Ar^+^ ion sputtering, and all binding energies were calibrated with respect to the C 1s peak at 284.8 eV. Interfacial wettability of the surface toward the electrolyte was assessed by contact angle measurements (Phoenix300 Touch 140318, SEO, Korea). For operando OM analysis, images were acquired using a CMOS camera mounted on the LV150N microscope, while electrochemical measurements were conducted using a potentiostat (ZIVE SP1, WonATech, Korea) with a lab‐made cell specifically designed to enable real‐time imaging during electrochemical reactions. Operando OM images were quantitatively analyzed using ImageJ (NIH, USA). An identical analysis area was used for all images. A fixed threshold was applied under identical conditions, and the thresholded dark‐area fraction was calculated as the ratio of the thresholded area to the total analysis area. 3D morphology of the Zn‐deposited electrodes was examined by LSCM (LSM 700, Carl Zeiss, Germany).

### 4.4 Electrochemical Characterizations

Aqueous 2 m ZnSO_4_ electrolyte and glass fiber separator (GF/F, Whatman) were used for electrochemical measurements. Electrochemical cells were assembled in CR2032‐type coin cell configurations, and all cells were prepared at room temperature. Galvanostatic charge and discharge cycling was performed using battery cyclers (BCS3000Le32, WonATech, Korea; CT‐4008T, Neware, China). CV were acquired within a voltage window of −0.2 to 0.4 V at a scan rate of 0.2 mV s^−1^. CA was performed at an applied voltage of −150 mV. Tafel polarization curves were recorded over a voltage range of −0.25 to 0.25 V at a scan rate of 1 mV s^−1^. The apparent *i*_0_ was estimated from Zn||Zn symmetric‐cell rate tests (Figure 3f) by linearly fitting the relationship between the total voltage hysteresis and the applied current density. Symmetric cells were sequentially cycled at current densities of 0.5, 1, 2, and 4 mA cm^−2^ under identical areal capacities. For each current density, the total voltage hysteresis was determined from the difference between the steady‐state plating and stripping voltage plateaus. The average voltage hysteresis was calculated from 10 consecutive plating/stripping cycles, and the corresponding standard deviations were obtained. The apparent *i*_0_ was estimated according to

$$i\approx i_{0}\frac{F}{2RT}\eta$$

where *F* is the Faraday constant, *R* is the gas constant, *T* is the absolute temperature, and *η* represents the total voltage hysteresis. The calculated *i*_0_ values were used as comparative kinetic parameters for evaluating the relative Zn plating/stripping behavior of different electrodes under identical measurement conditions. For full‐cell evaluation, NH_4_V_4_O_10_ was designed as a cathode material [49]. As‐prepared NH_4_V_4_O_10_ powder was mixed with Super‐P as a conductive additive and poly(vinylidene fluoride) (PVDF) as a binder at a weight ratio of 7:2:1, using N‐methyl‐2‐pyrrolidone (NMP) as the solvent to form a homogeneous slurry. The resulting slurry was coated onto a stainless‐steel foil and dried in a vacuum oven at 100°C for 24 h. The average mass loading of NH_4_V_4_O_10_ cathode was approximately 2.5 mg cm^−2^. The full cell, consisting of the laser‐treated electrode as an anode and the synthesized NH_4_V_4_O_10_ as a cathode, was evaluated over a voltage range of 0.4–1.4 V at a specific current of 2 A g^−1^. Prior to galvanostatic charge and discharge cycling, Zn^2+^ pre‐intercalation was carried out by applying a calculated amount of charge to achieve the desired Zn content (0.1, 0.2, and 0.5 Zn per formula unit) as shown in Figure S31. CVs of the full cells were acquired within a voltage window of −0.4 to 1.4 V at a scan rate of 0.2 mV s^−1^.

### 4.5 Statistical Analysis

For the apparent *i*_0_ analysis, the total voltage hysteresis at each applied current density was determined from Zn||Zn symmetric‐cell rate measurement. For each current density (0.5, 1, 2, and 4 mA cm^−2^), the median voltage hysteresis was extracted from the plateau region (0.15–0.85 h) of 10 individual plating/stripping half‐cycles, and the values are presented as mean ± standard deviation (SD, *n* = 10). The apparent *i*_0_ was estimated by linear fitting of the relationship between the mean voltage hysteresis and the applied current density. Quantitative roughness analysis was performed using representative height profiles extracted from the LSCM images. The arithmetic average roughness (R_a_) values are presented as mean ± SD, with *n* = 11 independent height profiles for each sample. No statistical significance test was performed because the data were used for descriptive comparison.

## Author Contributions

**Seyoung Han**: data curation, investigation, writing – original draft. **Taehyun Lee**: writing – original draft, visualization, data curation. **Haeun Kim**: investigation. **Ji Hyun Um**: conceptualization, writing – review and editing, supervision, project administration. **Dongkyoung Lee**: project administration, supervision, resources, writing – review and editing.

## Funding

This work was supported by the National Research Foundation of Korea (NRF) grant funded by the Korea government (MSIT) (No. RS‐2023‐00210422). The research described herein was supported by the National Research Foundation of Korea (NRF) (RS‐2023‐00208039) funded by the Ministry of Science and ICT (MSIT), Republic of Korea. This work was supported by the Korea Institute of Energy Technology Evaluation and Planning (KETEP) and the Ministry of Climate, Energy & Environment (MCEE) of the Republic of Korea (No. RS‐2024‐00394769) and was supported by the Korea Institute for Advancement of Technology (KIAT) (RS‐2026‐25586201) funded by the Ministry of Trade, Industry, and Resources (MOTIR, Korea).

## Conflicts of Interest

The authors declare no conflicts of interest.

## Supporting information




**Supporting File 1**: advs77286‐sup‐0001‐SuppMat.docx.

**Supporting File 2**: advs77286‐sup‐0002‐MovieS1.mp4.

**Supporting File 3**: advs77286‐sup‐0003‐MovieS2.mp4.

**Supporting File 4**: advs77286‐sup‐0004‐MovieS3.mp4.

**Supporting File 5**: advs77286‐sup‐0005‐MovieS4.mp4.

**Supporting File 6**: advs77286‐sup‐0006‐MovieS5.mp4.

## Data Availability

All data supporting the findings of this study are available within the article and its Supporting Information. Additional data are available from the corresponding author upon reasonable request.
