# Supplementary material for: Geometry‐Regulated Electrode‐Wide Nucleation Patterns in Aqueous Zinc Anodes
Source: Adv Sci (Weinh). 2026 Sep 6:e77286. Online ahead of print. doi: 10.1002/advs.77286 (PMC13547539; doi:10.1002/advs.77286)
Supplement: Supplementary file 1 [file ADVS-9999-e77286-s001.docx]

Supporting Information

**Geometry-Regulated Electrode-Wide Nucleation Patterns in Aqueous Zinc Anodes**

*Seyoung Han, Taehyun Lee, Haeun Kim, Ji Hyun Um,^*^ and Dongkyoung Lee^*^*

S. Han,^†^ H. Kim, Prof. J. H. Um, Prof. D. Lee

Department of Future Convergence Engineering, Kongju National University, Cheonan 31080, Republic of Korea

E-mail: jhum@kongju.ac.kr

E-mail: ldkkinka@kongju.ac.kr

T. Lee,^†^ Prof. J. H. Um

Department of Chemical Engineering, Kongju National University, Cheonan 31080, Republic of Korea

Prof. J. H. Um

Center for Future Sustainable Technology, Kongju National University, Cheonan 31080, Republic of Korea

Prof. J. H. Um, Prof. D. Lee

Global Institute of Manufacturing Technology (GITECH), Kongju National University, Cheonan 31080, Republic of Korea

Prof. D. Lee

Department of Mechanical and Automotive Engineering, Kongju National University, Cheonan 31080, Republic of Korea

Prof. D. Lee

Department of Semiconductor Engineering, Kongju National University, Cheonan 31080, Republic of Korea

^†^These authors equally contributed to this work.

**Figure S1.** a) OM images of laser-patterned Zn foil with increasing hole opening ratio from 1% to 11%. The OM image was acquired at 10x magnification; b) Corresponding quantitative relationship between the hole opening ratio and hole density.

**Figure S2.** SEM images of laser-patterned Zn foil with varying hole opening ratios (1, 5, 9, and 11), shown at different magnifications.

**Figure S3.** Contact angle measurements of 2 M ZnSO_4_ electrolyte on bZn and L1, L5, L9, and L11 electrodes. The black line and inset images represent the contact angle measured 1 s after droplet deposition, whereas the orange line denotes the saturated contact angle. The laser-patterned electrodes exhibit progressively reduced contact angles with increasing hole opening ratio, indicating enhanced wettability. This behavior is attributed to the porous surface morphology induced by laser structuring, which increases the effective electrode-electrolyte contact area.

**Figure S4.** a) OM and b) SEM images of L9 and bZn electrodes at different magnifications.

**Figure S5.** XPS depth profiles of L9 and bZn, with a) survey, b) O 1s, and c) Zn 2p spectra.

**Figure S6.** a) Time-voltage profiles of bZn and laser-patterned Zn||Cu asymmetric cells at a current density of 1 mA cm^-2^ with a capacity of 1 mAh cm^-2^; b) Enlarged view of the initial plating region highlighted in a), showing differences in nucleation overpotential among the electrodes.

**Figure S7.** CE of L9||Cu asymmetric cell at a current density of 1 mA cm^-2^ with a capacity of 1 mAh cm^-2^.

**Figure S8.** a) Time-voltage profiles of L9||Cu asymmetric cell at a current density of 1 mA cm^-2^ with a capacity of 2 mAh cm^-2^; b) Time-voltage profiles of L9||Cu under stepwise areal capacity conditions (1 and 2 mAh cm^-2^) at a fixed current density of 1 mA cm^-2^; c) Extracted voltage profiles of L9||Cu at ~204 and ~404 h, recorded at areal capacities of 1 and 2 mAh cm^-2^, showing consistent plating/stripping behavior with limited polarization.

**Figure S9.** CV curves of bZn and laser-patterned Zn||Cu asymmetric cells at a scan rate of 0.2 mV s^-1^.

**Figure S10.** Tafel polarization curves of bZn, L5, L9, and L11 electrodes with the corresponding corrosion current densities.

**Figure S11.** Locally magnified voltage profiles of the L9 symmetric cell at 500, 1000, 2000, and 3000 h.

**Figure S12.** Zn||Zn symmetric-cell cycling performance of bZn and L9 electrodes at a current density of 5 mA cm^-2^ with a capacity of 10 mAh cm^-2^, which corresponds to a DOD of approximately 36.4% for the laser-patterned L9 electrode.

**Figure S13.** a) and b) Capacity-voltage profiles of L9 and bZn electrodes at a current density of 1 and 5 mA cm^-2^ under a fixed capacity of 1 mAh cm^-2^; SEM images of Zn deposition obtained at different current densities of c) L9 and d) bZn electrodes.

**Figure S14.** a) Comparison of the total voltage hysteresis of bZn and L9 electrodes obtained from Zn||Zn symmetric-cell rate measurement at different current densities (Figure 3f). Error bars represent the standard deviation calculated from 10 plating/stripping cycles at each current density; b) Estimation of the apparent *i*_0_ by linearly fitting the relationship between the total voltage hysteresis and current density.

**Figure S15.** a) Time-voltage profiles during Zn plating at increasing areal capacities; b) *Ex situ* SEM images at corresponding areal capacities (0, 0.25, 0.5, and 1 mAh cm^-2^) at a current density of 1 mA cm^-2^.

**Figure S16.** a) Time-voltage profile and b) *operando* OM images of L9 at a current density of 1 mA cm^-2^.

**Figure S17.** Time-voltage profile and *operando* OM images of L5 during the first plating, first stripping, and second plating at 2 mA cm^-2^.

**Figure S18.** Time-voltage profile and *operando* OM images of L9 during the first plating, first stripping, and second plating at 2 mA cm^-2^.

**Figure S19.** Time-voltage profile and *operando* OM images of L11 during the first plating, first stripping, and second plating at 2 mA cm^-2^.

**Figure S20.** Time-voltage profile and *operando* OM images of bZn during the first plating, first stripping, and second plating at 2 mA cm^-2^.

**Figure S21.** *Operando* OM images of L5, L9, L11, and bZn electrodes at different stages of the 1^st^ Zn plating (0-3 mAh cm^-2^). Yellow highlighted regions indicate areas of pronounced Zn accumulation, and the symmetric pink circles indicate bubbles.

**Figure S22.** *Operando* OM images of L5, L9, L11, and bZn electrodes at different stages of the 1^st^ Zn plating (3 mAh cm^-2^), 1^st^ stripping (0-3 mAh cm^-2^), and 2^nd^ plating (3 mAh cm^-2^).

**Figure S23.** OM images of L9 electrode plated to 3 mAh cm^-2^ at a current density of 2 mA cm^-2^.

**Figure S24.** a) 3D LSCM height maps of the L9 and bZn electrodes after Zn plating at a current density of 2 mA cm^-2^ with a capacity of 2 mAh cm^-2^; b) Representative height profiles extracted from the LSCM height maps shown in a). The corresponding arithmetic average roughness (R_a_) values were calculated from 11 height profiles extracted at approximately 30 μm intervals across the 300 × 300 μm scanning area (mean ± SD, *n* = 11).

**Figure S25.** Post-cycling OM and SEM images of a) L5, L9, and L11 electrodes after 25 cycles and b) L9 electrode after 50 cycles (1 mA cm^-2^, 1 mAh cm^-2^).

**Figure S26.** a) Low-magnification cross-sectional FIB-SEM image of the L9 electrode after 25 Zn plating/stripping cycles; b) Higher-magnification cross-sectional FIB-SEM image of the blue boxed region in a). The hole interior is occupied by newly deposited lamellar Zn, while the yellow boxed regions indicate the original hole-edge regions.

**Figure S27.** XRD patterns of pristine and cycled bZn and L9 electrodes. All remaining diffraction peaks were assigned to metallic Zn.

**Figure S28.** CV curves of a) L9||NVO and b) bZn||NVO full cells during 8 cycles.

**Figure S29.** Initial capacity-voltage profiles during pre-intercalation process. The extent of Zn^2+^ pre-intercalation was controlled by applying a calculated charge based on the mass loading and target Zn stoichiometry (0.1, 0.2, and 0.5 per formula unit), yielding Zn_0.1_NVO, Zn_0.2_NVO, and Zn_0.5_NVO.

**Figure S30.** Capacity retention of bZn||NVO and bZn||Zn_0.2_NVO full cells at a current density of 2 A g^-1^, with average retention values calculated over 600 cycles.

**Figure S31.** Cycling performance and corresponding CE of L9||Zn_0.1_NVO, L9||Zn_0.2_NVO, and L9||Zn_0.5_NVO full cells at a current density of 2 A g^-1^.

**Figure S32.** Cycling performance and corresponding CE of L9||Zn_0.2_NVO full cells at a current density of 1 and 2 A g^-1^.

**Figure S33.** Cycling performance and corresponding CE of L9||Zn_0.2_NVO full cell at a current density of 3 A g^-1^.

**Figure S34.** SEM images of a) L9 and b) bZn anodes in full cells after 10 and 25 cycles at a current density of 1 A g^-1^, acquired at different magnifications. Each row corresponds to a specific electrode and cycle number.

**Figure S35.** Schematic illustration of laser patterning setup and the corresponding irradiation pattern on Zn foil, defining the hole distance.

**Figure S36.** Dependence of hole width on laser power across different processing regions.

**Figure S37.** Photographs (front side and back side) and OM images of the Zn foil processed at a) 6 W and b) 12 W.

**Table S1** The comparison of the laser-patterned Zn symmetric cell with recently reported 3D-structured Zn anodes.

| Electrode structure | Current density  (mA cm^-2^) | Plating/strip-  ping time (h) | Cycle life (h) | Reference |
| --- | --- | --- | --- | --- |
| 3D laser-patterned Zn | 0.5 | 1 | 3400 | This work |
| Hydrophobic micropatterned Zn | 1 | 0.5 | 1000 | 1 |
| High-aspect-ratio  hole-array Zn | 1 | 1 | 2400 | 2 |
| 3D microhole Zn | 1 | 1 | 1000 | 3 |
| Micro-nano textured Zn | 4 | 0.5 | 1400 | 4 |
| Crystal-faceted  ultra-thin Zn | 1 | 0.25 | 2428 | 5 |
| 3D finned Zn | 1 | 0.5 | 2680 | 6 |
| 3D pore-patterned Zn | 1 | 1 | 4000 | 7 |
| Honeycomb-structured Zn | 5 | 0.2 | 2000 | 8 |
| (002)-exposed 3D Zn | 5 | 0.2 | 6000 | 9 |
| Crystal-faceted Zn | 0.2 | 1 | 2200 | 10 |
| 3D etched Zn | 0.1 | 1 | 250 | 11 |
| Microgrooved Zn | 0.5 | 1 | 900 | 12 |
| Crystal-faceted microgrooved Zn | 1 | 1 | 550 | 13 |
| Grain-boundary-rich Zn | 1 | 1 | 1132 | 14 |
| Crystal-faceted porous Zn | 0.5 | 0.5 | 1600 | 15 |
| (002)-oriented  sharp-edge Zn | 3 | 1/3 | 250 | 16 |
| Microcracked Zn | 1 | 1 | 400 | 17 |
| Hierarchical micro/nanostructured Zn | 2 | 0.5 | 2530 | 18 |
| Mesh-grooved Zn | 0.5 | 1 | 400 | 19 |

**Table S2** Laser parameters in this study.

| Laser parameter | Value |
| --- | --- |
| Power | 6 - 20 W |
| Pulse duration | 4 ns |
| Repetition rate | 500 kHz |
| Number of pulse | 200 pulses |
| Wavelength | 1064 nm |
| Spot diameter | 30 μm |

**References**

1.	B. Xu, Y. Liao, S. To, et al., “Micro-Hydrophobic Microstructure Balance Electric Field Distribution and Wettability for Ultra-Stable Zinc Anodes,” *Advanced Energy Materials* 16 (2026): e71039, <https://doi.org/10.1002/aenm.71039>.

2.	J. Cao, X. Rao, Y. Zhang, et al., “Anisotropic Chemical Etching toward Cavitated and Crystal-Faceted Zinc Anodes with Enhanced Electrochemical Kinetics and Stability,” *Energy Storage Materials* 84 (2026): 104762, <https://doi.org/10.1016/j.ensm.2025.104762>.

3.	X. Liu, R. Wu, X. Hu, et al., “3D Porous Zinc Scaffold Anodes for Enhanced Stability and Performance in Zinc-Ion Energy Storage Systems,” *ACS Nano* 19 (2025): 26147–26160, <https://doi.org/10.1021/acsnano.5c07729>.

4.	S. Zhan, Z. Liu, F. Ning, et al., “Structure Engineering by Picosecond Laser Lithography Boosts Highly Reversible Zn Anode,” *Advanced Functional Materials* 35 (2025): 2417546, <https://doi.org/10.1002/adfm.202417546>.

5.	Y. Lin, X. Li, Y. Yang, et al., “Ultra-Thin Zn with Preferential Crystal Plane for Aqueous Zinc-Metal Batteries,” *Journal of Energy Storage* 128 (2025): 117188, <https://doi.org/10.1016/j.est.2025.117188>.

6.	H.-Y. Chen, H.-I. Tsai, T.-Y. Lin, et al., “Realization of Protective-Layer-Free Zn Anode by Three-Dimensional Architectural Design toward High Performance Aqueous Zinc Ion Batteries,” *Journal of Energy Storage* 134 (2025): 118234, <https://doi.org/10.1016/j.est.2025.118234>.

7.	X. Fu, L. Wang, G. Li, et al., “Constructing 3D Zinc Anode by Acid Etching Strategy toward Long-Cycling Aqueous Zinc-Ion Batteries,” *Electrochimica Acta* 536 (2025): 146800, <https://doi.org/10.1016/j.electacta.2025.146800>.

8.	D. Meng, X. Liang, Q. Liu, et al., “An Electrostripping Strategy for Constructing a 3D Honeycomb-Like Zn Anode toward Dendrite-Free Zinc-Ion Batteries,” *Advanced Functional Materials* 34 (2024): 2411047, <https://doi.org/10.1002/adfm.202411047>.

9.	X. Chen, Z. Zhai, T. Yu, et al., “Constructing a 3D Zinc Anode Exposing the Zn(002) Plane for Ultralong Life Zinc-Ion Batteries,” *Small* 20 (2024): 2401386, <https://doi.org/10.1002/smll.202401386>.

10.	D. Xu, B. Chen, X. Ren, et al., “Selectively Etching-Off the Highly Reactive (002) Zn Facet Enables Highly Efficient Aqueous Zinc-Metal Batteries,” *Energy & Environmental Science* 17 (2024): 642–654, <https://doi.org/10.1039/D3EE02522E>.

11.	S. Gull, C.-Y. Lai, W.-H. Lu, et al., “Beyond Conventional: Unveiling the Impact of Zn Anode Pretreatment in Aqueous Zinc-Ion Batteries,” *Journal of Materials Chemistry A* 12 (2024): 28919–28929, <https://doi.org/10.1039/D4TA03160A>.

12.	B.-B. Sui, L. Sha, P.-F. Wang, et al., “Anode Surface Pretreatment to Optimize Electrochemical Performance of Aqueous Zinc-Ion Batteries,” *Solid State Ionics* 405 (2024): 116453, <https://doi.org/10.1016/j.ssi.2024.116453>.

13.	B.-B. Sui, W.-J. Song, L. Sha, et al., “Citric Acid Etched Zinc Anode Surface to Improve the Stability of Aqueous Zinc-Ion Battery,” *Solid State Ionics* 405 (2024): 116437, <https://doi.org/10.1016/j.ssi.2023.116437>.

14.	S. Lian, Z. Cai, M. Yan, et al., “Ultra-High Proportion of Grain Boundaries in Zinc Metal Anode Spontaneously Inhibiting Dendrites Growth,” *Angewandte Chemie International Edition* 63 (2024): e202406292, <https://doi.org/10.1002/anie.202406292>.

15.	L. Chen, F. Liu, Y. Dong, et al., “Anion-Induced Facet-Selective Etching of Zn Metal Anode for Long Cycle Aqueous Zn-Ion Batteries,” *Journal of Energy Storage* 87 (2024): 111529, <https://doi.org/10.1016/j.est.2024.111529>.

16.	H. B. Jeong, D. I. Kim, G. Yoo, et al., “Selective Control of Sharp-Edge Zinc Electrodes with (002) Plane for High-Performance Aqueous Zinc-Ion Batteries,” *Journal of Materials Chemistry A* 12 (2024): 15265–15277, <https://doi.org/10.1039/D4TA01013B>.

17.	L. Sha, B.-B. Sui, P.-F. Wang, et al., “Micro-Cracking on the Surface of Zinc Metal Improves the Cycle Performance of Aqueous Zinc Ion Batteries,” *Journal of Energy Storage* 90 (2024): 111843, <https://doi.org/10.1016/j.est.2024.111843>.

18.	Y. Liu, Y. Ding, Z. Liu, et al., “Ultrafast Laser One-Step Construction of 3D Micro-/nanostructures Achieving High-Performance Zinc Metal Anodes,” *PhotoniX* 5 (2024): 6, <https://doi.org/10.1186/s43074-024-00122-x>.

19.	L. Sha, B.-B. Sui, P.-F. Wang, et al., “Printing 3D Mesh-Like Grooves on Zinc Surface to Enhance the Stability of Aqueous Zinc Ion Batteries,” *Journal of Colloid and Interface Science* 647 (2023): 421–428, <https://doi.org/10.1016/j.jcis.2023.05.171>.
